# Examination of National Basketball Association (NBA) team values based on dynamic linear mixed models

**DOI:** 10.1371/journal.pone.0253179

**Published:** 2021-06-17

**Authors:** Efehan Ulas

**Affiliations:** Faculty of Science, Department of Statistics, Cankiri Karatekin University, Cankiri, Turkey; Sapienza University of Rome, ITALY

## Abstract

In the last decade, NBA has grown into a billion-dollar industry where technology and advanced game plans play an essential role. Investors are interested in research examining the factors that can affect the team value. The aim of this research is to investigate the factors that affect the NBA team values. The value of a team can be influenced not only by performance-based variables, but also by macroeconomic indicators and demographic statistics. Data, analyzed in this study, contains of game statistics, economic variables and demographic statistics of the 30 teams in the NBA for the 2013–2020 seasons. Firstly, Pearson correlation test was implemented in order to identify the related variables. NBA teams’ characteristics and similarities were assessed with Machine Learning techniques (K-means and Hierarchical clustering). Secondly, Ordinary linear regression (OLS), fixed effect and random effect models were implemented in the statistical analyses. The models were compared based on Akaike Information Criterion (AIC). Fixed effect model with one lag was found the most effective model and our model produced consistently good results with the R^2^ statistics of 0.974. In the final model, we found that the significant determinants of team value at the NBA team level are revenue, GDP, championship, population and key player. In contrast, the total number of turnovers has a negative impact on team value. These findings would be beneficial to coaches and managers to improve their strategies to increase their teams’ value.

## Introduction

**Fig 1:**
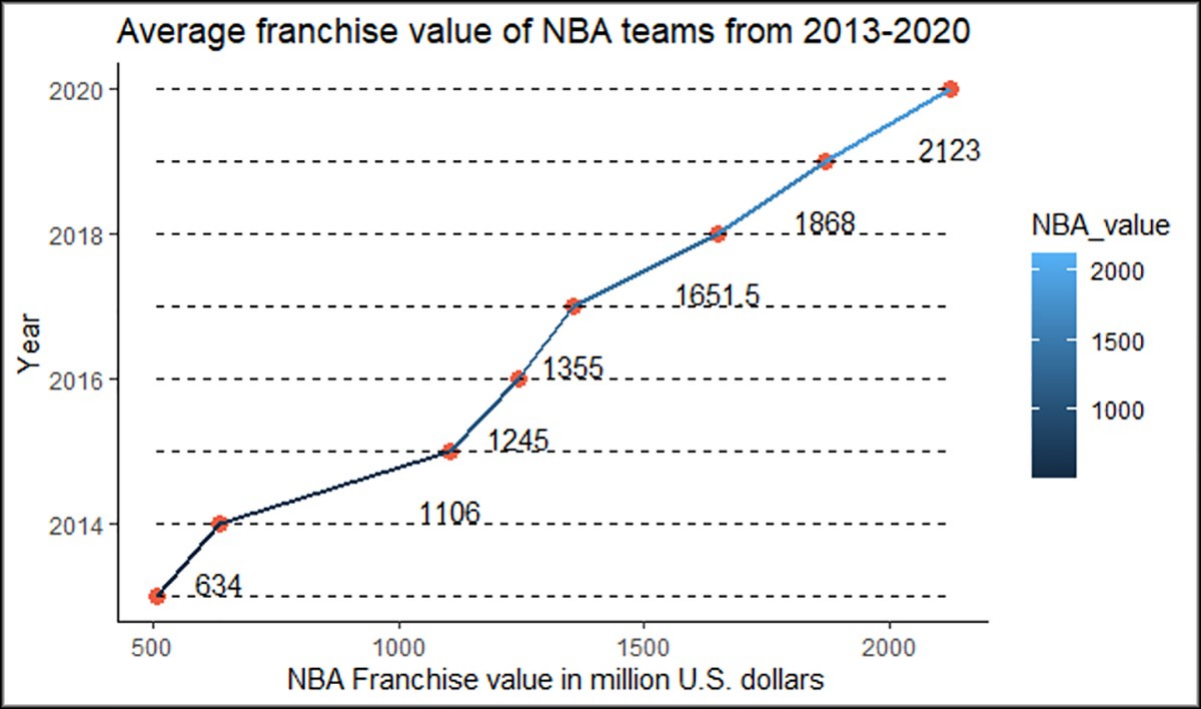
Average franchise value of NBA teams from 2013–2020.

The value of NBA teams has increased significantly in the last years. After 2014, the tremendous increase in the value of NBA franchises has attracted the attention of owners and researchers. NBA owners also have an interest in determinants of NBA team values. Although the NBA does not release detailed financial reports to the public, these financial reports can be obtained from Forbes reports and other sport websites. In the last seven years, franchise values of the NBA have grown around 30% from 2015 to 2020 (Fig 1). The reason behind the increase in the team values cannot be explained only by the significant performance statistics of the teams. Effective game performances of the teams do not always equal success in the team values. For example, New York Knicks has not made the playoffs since 2012, but is still the most valuable team in the NBA according to Forbes in 2020. Therefore, it is important to investigate other factors which may affect team values such as economic indicators, demographic statistics and financial variables.

The population and gross national product (GDP) in the city where the team is located may have a positive effect on the value of the team. For instance, the population of the city might positively affect basketball marketing. The higher population can help the sales of the souvenirs like team uniforms and other products. Similarly, the number of wealthier supporters is high in the metropolitan cities and this can help to increase team values directly. To our knowledge, there are only a few studies related to determinants of NBA franchise values and these studies do not considered NBA teams individually. [1] used panel data on NBA franchises for the years between 2009 and 2016 to determine significant indicators of franchise value in the NBA and the valuation approaches of the study predicted the franchise in Seattle to be $1.4 billion in 2017. [2] compared the determinants of firm values in the United States and Europe over the period of 2004–2011. They considered NBA, Major League Baseball (MBL), National Football League (NFL), National Hockey League (NHL) and European soccer values and found that determinants of team values in the USA were not the same as those in Europe. [3] examined the effect of team nomenclature, team relocation and stadiums on franchise values for NBA, MBL, NFL and NHL. They highlighted that team performance; market size and the new stadium rises the team value in the MLB but not in the NBA. However, moving from an old facility into a new one increases the values of NBA teams. [4] investigated the franchise values of American professional sports teams in the 3 national leagues. Their findings showed that new facilities were not effective in the increasing of franchise values of the NBA.

Many other studies consider the NBA panel data in order to analyze performances of the teams. Performance analysis in the NBA is evaluated by statistical estimation of available data. Thus, effective parameters need to be determined in order to analyze the panel data. Furthermore, performance indicators may have effect on team values. Therefore, it is important to understand the studies related to performance analysis of game dynamics. In basketball, technical and physical performances are considered as the most important influences on the team performance during a match [5]. In addition, offensive factors determine sport performance in the NBA [6]. Coaches consider the quality of opposition when playing against stronger and weaker opponents [7]. Thus, such game plans may reduce or increase the total game statistics of the teams. Although many studies indicated the importance of game performance, its effect on the team values has not been examined in detail. Therefore, the goal of this paper is to examine the game performance statistics, economic indicators and demographic parameters that significantly affect the team values based on the dynamic linear panel regression models. In this study, the game performance statistics, economic indicators and demographic parameters and logarithmic transformation of some of the variables has been considered in order to estimate the team values in the models.

Parameter selection plays a critical role in estimating the team values. In this study, the performance statistics and economic indicators used to estimate team values were chosen according to literature. Papers included population [8,9], GDP [10,11], total assists [12,13], revenue [14], allstar [15], winning percentage [16], championship [17], total points [18], total turnover [19], home attendance [20], key player [1,21], team value [1,2] in their statistical analysis. Therefore, we considered the variables that can potentially affect the team values by evaluating the literature. In addition, it would be misleading to use only performance variables when determining the value of the NBA teams. Variables such as the population and wealth of the city, the average supporter capacity, revenue and the team popularity should be considered in order to understand the sharp increase in the last years.

In literature, examination of professional sports franchise value was rare before the 2000s. The reason behind this can be explained as the owners of professional sport teams are wealthy enough to sustain losses [22]. After the 2000s, many studies have been published about professional sports franchise values for all professional sports leagues such as MLB, NFL and NHL. These studies can be basically divided into two categories: modelling franchise values by using Forbes reports and examining franchise values from historical growth rates. This is not the case in this research because we do not only focus on franchise value but also on team values individually. Also, analyzing only performance indicators produces low prediction rates for team values. Therefore, economic parameters and demographic variables are included in the analysis. First of all, the data are combined from different sources for the NBA seasons between 2013–2020. Correlation analysis has been implemented to the variables in order to understand the relation between those variables. Then, similarities between NBA teams are identified based on the selected variables by using Machine Learning techniques, K-means clustering and Hierarchical clustering. Thus, besides determining the parameters that affect the team value by using linear mixed models, we aimed to investigate the NBA teams individually and evaluate their similarities with each other. Afterward, based on the selected outcomes, three different dynamic linear models; OLS, fixed effect and random effect models are implemented. The lag value of the dependent variable is considered in the models in order to reduce the correlation problem. The final model is selected based on AIC scores [23]. Therefore, the aim of this study is to (i) investigate the independent and interactive effects of team performance statistics, economic and demographic indicators on the team values and (ii) to examine these indicators and performance parameters, that significantly affect the team value, through a case study of the NBA teams using suitable statistical and machine learning methods.

## Materials and methods

### Data

The data consist of 13 economic and demographic variables and performance parameters that indicate the characteristic of NBA teams in the National Basketball League for the 2013–2020 season. In the dataset, the variables and performance indicators are related to the values of the teams, not to the individual values of the players. The dependent variable, team values, is the variable we aim to estimate by using the remaining 12 variables. The variables which are used in the analysis and their definition are given in Table 1. All the observations are collected from Forbes, census, opendatanetwork and NBA websites. The detailed list of the websites from where the data is collected is given in the Table 2.

**Table 1:** Variables and their descriptions.

Variable	Description
Team_Value	Annual value of the team: Continues variable
Revenue	Annual revenue of the team: Continues variable
Win_Percent	Winning percentage of the team in a season
Assist	Average assists of the team per game
Coast	The coast of the team: ***east*** or ***west***
Turnover	Average turnover of the team per game
Point	Average points of the team per game
Championship	Total number of championships
Population	Population of the team city
GDP	Gross domestic production per capita of the team city
Home_Attendence	Average number of home attendence in the stadium
Allstar	Number of all-stars of the team in that year
Point per game	Average point per game
Key Player	The number of highest-paid NBA players for each team

**Table 2:** The list of the data sources.

Variable	Source of the Variable
*Assist*	https://www.espn.com/nba/stats/team
*Turnover*	https://www.espn.com/nba/stats/team
*Team Value*	https://www.sportico.com/valuations/teams and https://www.forbes.com/sites/kurtbadenhausen
*Points*	https://www.espn.com/nba/stats/team
*Cost*	https://www.nba.com/
*Winning Percent*	https://www.espn.com/nba/stats/team
*Allstar*	https://www.nba.com/
*Championship*	https://www.landofbasketball.com/championships/summary_of_winners.htm
*Population*	https://worldpopulationreview.com/states
*GDP*	https://www.bea.gov/data/gdp/gdp-county-metro-and-other-areas
*Home Attendence*	http://www.espn.com/nba/attendance
*Key Player*	https://en.wikipedia.org/wiki/Highest-paid_NBA_players_by_season
*Revenue*	https://www.statista.com/statistics/193467/total-league-revenue-of-the-nba-since-2005/ and https://www.forbes.com/sites/kurtbadenhausen/2019/02/06/nba-team-values-2019-knicks-on-top-at-4-billion/?sh=416b0861e667

Some variables were directly taken, while others were derived from the existing ones by using mathematical transformation with the goal of supplying different and potentially more advantageous, sensible and insightful knowledge. In this study, the derived variables are as follows:

- GDP = Logarithmic transformation of gross domestic product per capita of the team city.
- Population = Logarithmic transformation of population of the team city
- Home Attendence = Logarithmic transformation of home team attendence (annual)

Since there are 30 teams in the NBA, 30x8 = 240 observations are included in the dataset. The estimation of some of the observations are replaced with missing values.

The list of the data sources is given below. The variables are collected from different sources and all list can be found in Table 2.

### Statistical approach

Basic statistical descriptors (mean and standard deviation (SD)) for the variables were calculated and given in Table 3.

**Table 3:** Summary of the variables.

Variable	Observation	Mean	S.D	Min	Max
Team Value(million$)	240	1314	823.93	312	4600
Assist	240	22.93	2.1418	18	30.4
Turnover	240	13.75	1.087	11	16.9
Points	240	104.6	6.1499	91.9	118.7
Win Percent	240	50.01	15.3228	12.2	89.02
Allstar	240	0.7917	0.84235	0	4
Championship	240	2.326		0	17
Key Player	240	0.333	0.604	0	3
Population	240	1657379	2086236	191697	8622698
GDP($)	240	59313	10635.75	41113	93687
Home Attendence	240	17742	1796.416	13487	21876
Revenue(million$)	240	220.9	75.4494	109	472
Coast	240				
Season	240				

After carefully examining the explanatory variables of the dataset, correlation test was implemented to capture if whether there is any relationship between variables. Afterwards, K-means clustering and hierarchical clustering approaches were applied in order to identify NBA team’s similarities based on selected variables. Finally, dynamic linear regression models were applied to investigate the variables that have the biggest influences on the team values. The unstandardized coefficients and standardized coefficients are calculated and compared in order to capture if the coefficients change due to different units. However, there was no remarkable difference between the coefficients of the both techniques. Three different significance levels; 0.1, 0.05, and 0.01 were considered in order to identify the statistically significant variables. The variables which are statistically significant based on the significance level are shown with different Latin letters. A *p* value less than selected three significance levels were considered to be statistically significant.

All the analyses were performed using R statistical software [24]. For correlation analysis, corr package [25] was used, while for illustration of the cluster analysis analysis”ggbiplot” [26] and for regression analysis”plm” package [27] were used.

## Methodology

Below is the formulation of fixed and random effects models.


$$Y_{it}=\beta_{0}+\beta_{1}X_{1,it}+\ldots +\beta_{k}X_{k,it}+\gamma_{2}E_{2}+\ldots +\gamma_{n}E_{n}+u_{it}$$ (1)

Here, *Y*_*it*_ is the dependent variable and *i* = entity and *t* = time. *X*_k,it_ indicates independent variables, *β*_*k*_ is the coefficient for the independent variables, *u*_it_ is the error term, *E*_*n*_ is the entity *n* and *γ*_2_ Is the coefficient. And time effect can be included to the entity effects model:

$$Y_{it}=\beta_{0}+\beta_{1}X_{1,it}+\ldots +\beta_{k}X_{k,it}+\gamma_{2}E_{2}+\ldots +\gamma_{n}E_{n}+\delta_{2}T_{2}+\ldots +\delta_{t}T_{t}+u_{it}$$ (2)

Where *T*_*t*_ is time as binary variable and *δ*_*t*_ is the coefficient for the binary time regressors. The formulation of the random effect model can be seen below.


$$Y_{it}=\beta X_{it}+\alpha +u_{it}+\varepsilon_{it}$$ (3)

Here, *Y*_*it*_ is the dependent variable and *i* = entity and *t* = time. *X*_k,it_ indicates independent variables, *u*_it_ is the between-error term and *ε*_*it*_ is the within-error term. In above formulations, it is assumed that $u_{it}∼N(0,\sigma_{u_{it}}^{2})$ is independent of $\varepsilon_{it}∼N(0,\sigma_{\varepsilon }^{2})$. In random effects models, our focus on the variance components, $\sigma_{u_{it}}^{2}$. Thus, group specific effects can be examined.

## Results

### Correlation analysis

**Fig 2:**
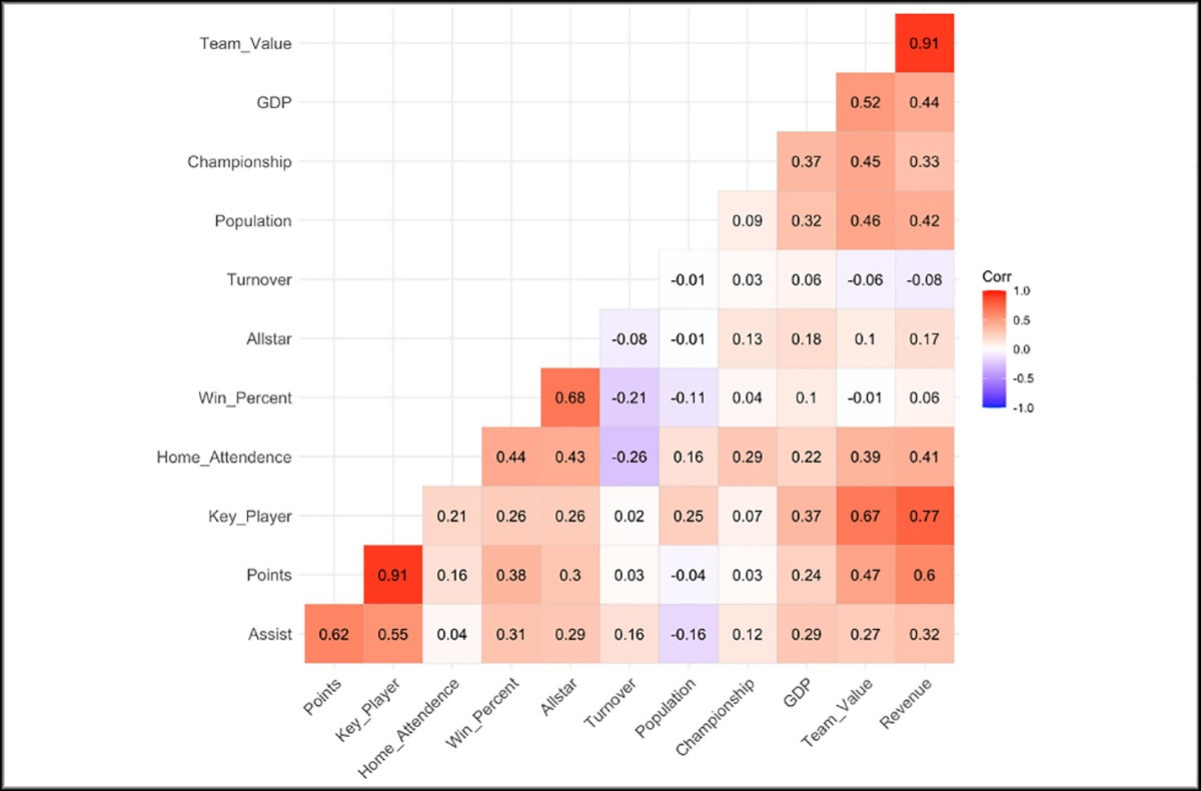
Correlation plot of the variables.

Correlation analysis is a useful approach for understanding the structure of the variables. It might be advantageous to consider correlation analysis before going through detailed statistical methods. Fig 2 illustrates the Pearson correlation scores of the continuous variables. The red boxes represent variables that have a positive relationship and blue boxes represents variables that have a negative relationship.

As expected, team value and revenue have a strong positive relationship (r = 0.91) which means that teams having higher revenue tend to have higher team value. Similarly, team value and key player have a strong positive relationship (r = 0.67). In contrast, home attendence and turnover have a negative relationship (r = -0.26) which means the high number of turnovers effects the number of home attendence in the teams. Unsurprisingly, winning percentage have a positive effect on the number of allstar. Since there are some independent variables that have correlation coefficient greater than 0.5, the multicollinearity was checked before going through the analysis. Therefore, variance inflation factor (VIF) was calculated in order to capture the multicollinearity. However, only the VIFs of the Point and Revenue were found around 3.5. All other variables change between 1–2. Therefore, we considered all these correlations in the modelling part because VIFs between 1 and 5 suggest that there is a moderate correlation, but it is not substantial enough to remove variables from the model [28]. The lag value of the dependent variable is included in the models which helps reducing the serial correlation problem.

**Fig 3:**
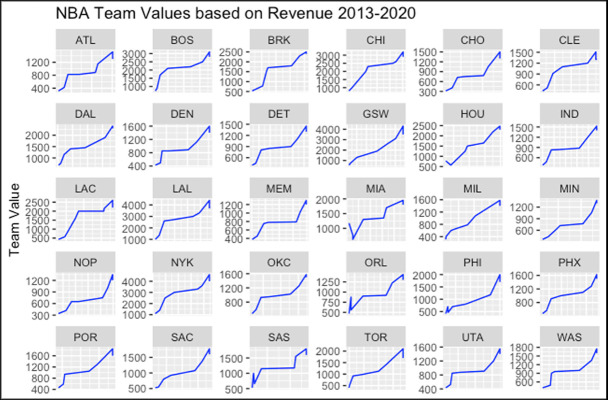
NBA team’s values vs revenues.

**Fig 4:**
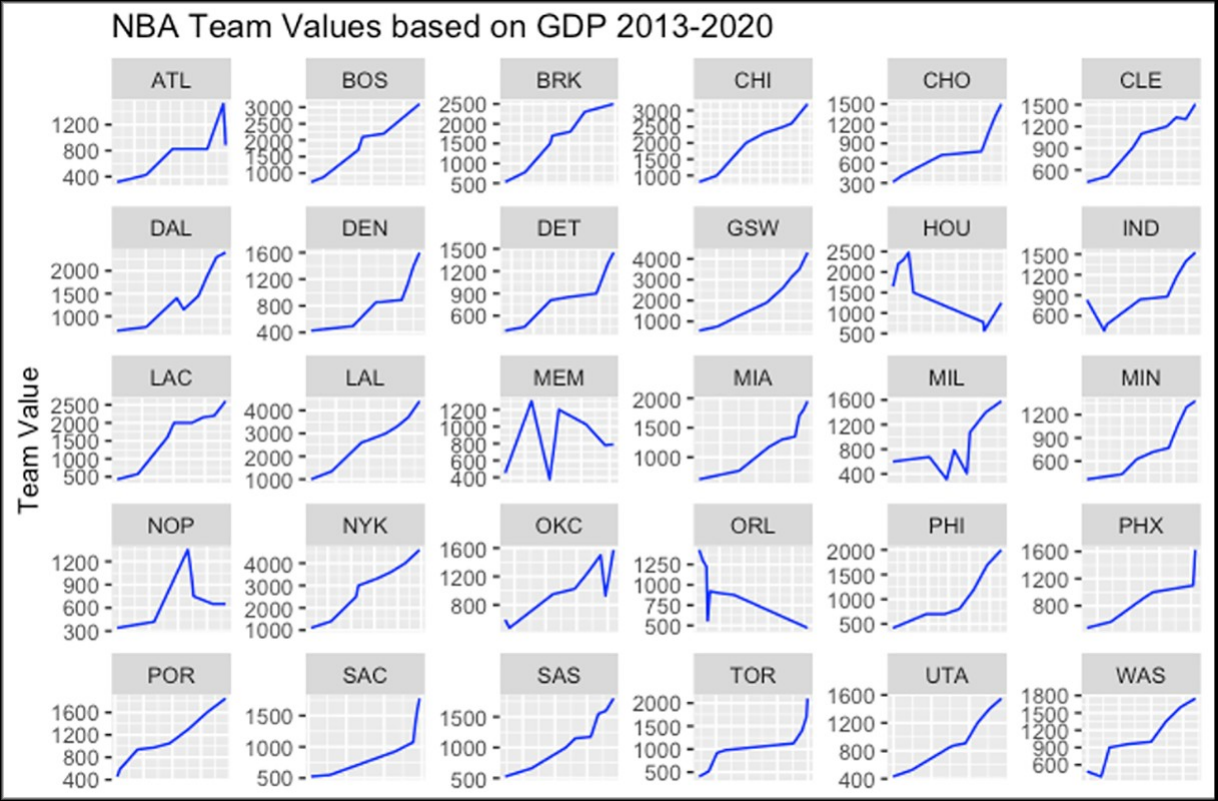
NBA team’s values vs GDP.

Fig 3 illustrates the relationship between NBA team values and revenue. In addition, Fig 4 shows the team values and GDP for each team respectively. As each team value increases, its revenue also increases. Although a similar scenario occurs in Fig 4, some of the GDPs reduced while the team’s value increased. This scenario is generally observed in cities with high numbers of immigrants.

It is interesting that the NBA might have an impact on the economy of the city. In Fig 4, the most of the team values have positive effect on the GDP. If the team value is increases, GDP is also increase for the most of the cities. However, if the value of the team decreases, it has neutral impact on the GDP. This might be due to the NBA brings in large amounts of money into the economy that affects both the team, and the city positively.

### Cluster analysis

**Fig 5:**
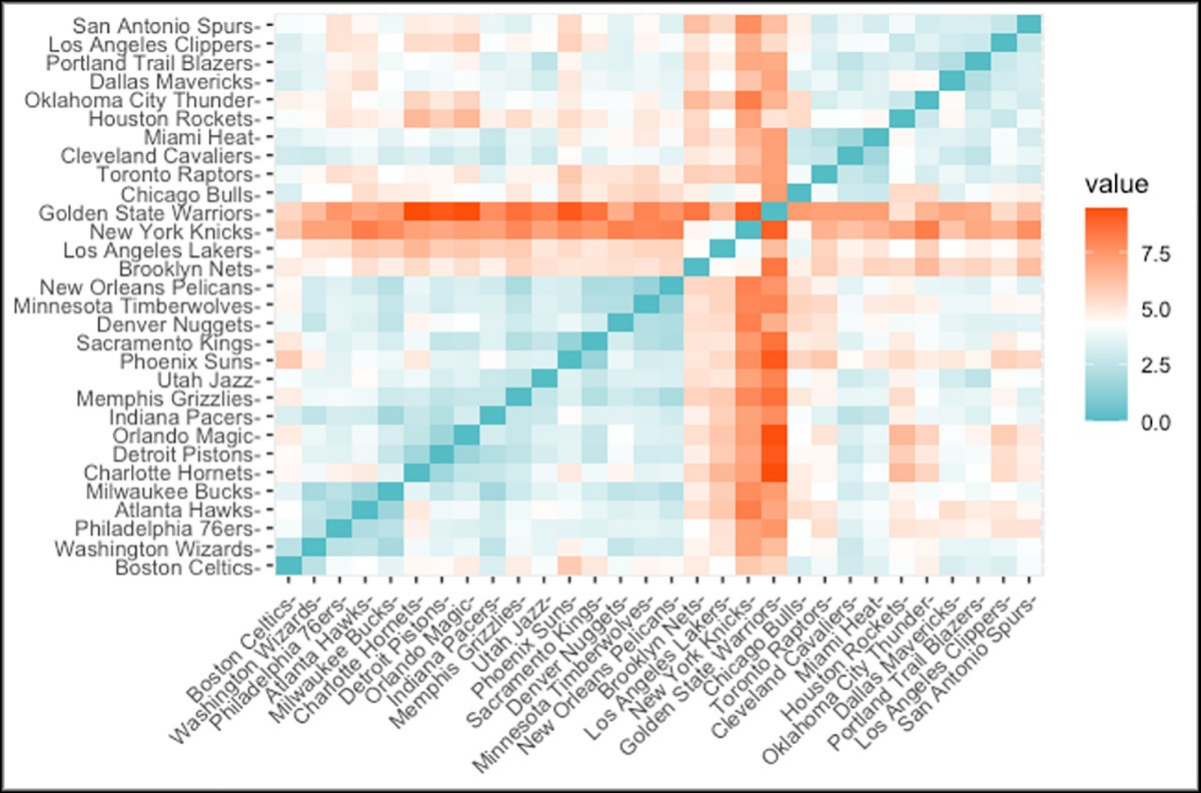
Distance matrix of the teams.

The aim of cluster analysis is to identify the internal grouping in a set of data. The data divided into k groups in the K-means clustering approach and each cluster is defined by its centroid. Similarities and dissimilarities of the teams are calculated with Euclidean distance in our analysis. This approach helps us to classify teams into groups. The outcome of the calculation is known as the distance matrix and it can be seen in the Fig 5 below.

High similarities and dissimilarities are shown with red and turquoise colors respectively in Fig 5. Teams whose revenue and the stadium capacity are more likely to have the least distance between them. The clustering is processed by minimizing the sum of the distances between each observation and the cluster centroid. The algorithm of the K-means clustering has four steps:

- Determining the number of clusters (k)
- Select randomly k teams from the data as an initial grouping centroid and each team of the data is assigned to its nearest centroid
- For each cluster, update the cluster centroid by generating the new mean values of all the team features
- Repeat step 2 and 3 until the cluster assignments are completed

**Fig 6:**
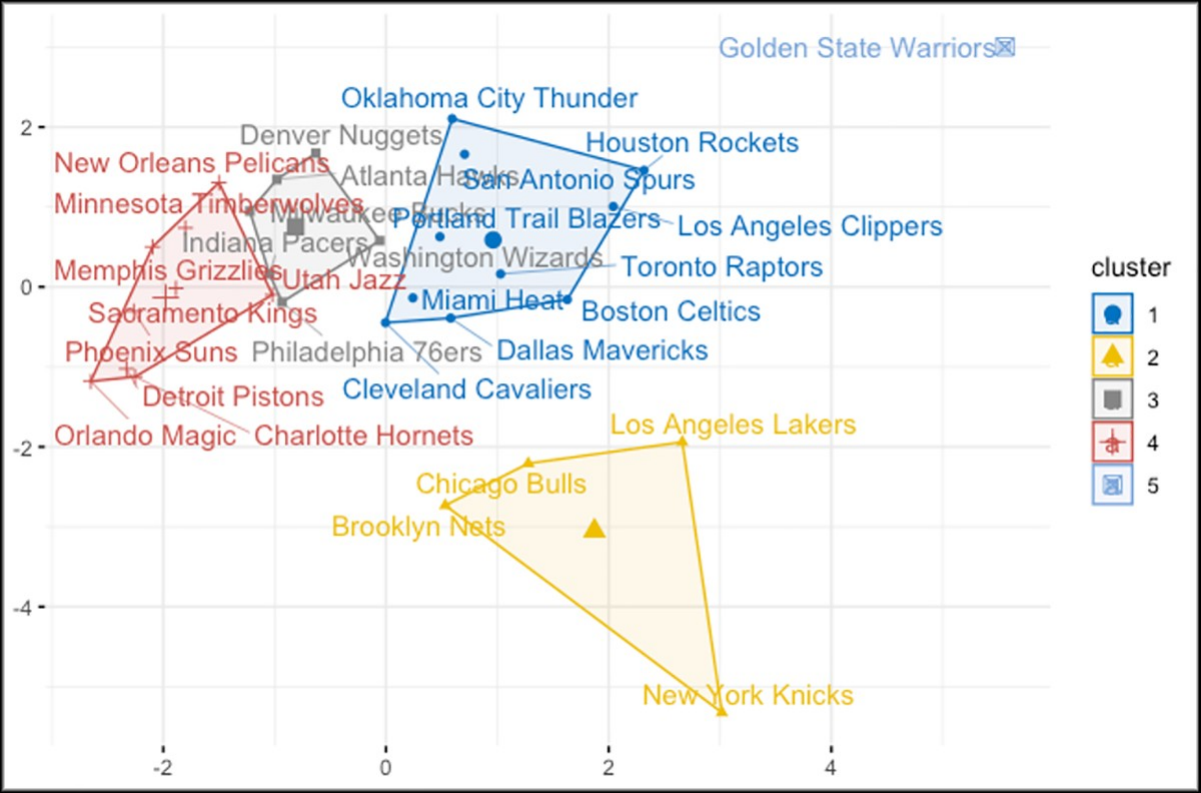
K-means clustering plot.

The optimal number of clusters are selected based on Gap statistics. The higher value of gap statistics was occurred for k = 5. Therefore, the number of clusters are selected as 5 where the gap statistic peaks and data are divided in to 5 groups based on k-means clustering. Fig 6 shows the final grouping of k-means clustering.

Light blue represents the Golden State Warriors. This team was separated from other teams and was assigned as a group alone. The most important reason for this outcome is that GSW played in the four NBA finals in the last six seasons and won the championship title 3 times. These outstanding statistics of GSW are the dominant reason that separates the team apart from the other teams in the data set. Therefore, we can call this group as the most successful team.

On the bottom of the plot (yellow color), there are teams with the most populated cities in the USA. The four teams in this group are those belonging to the three most populous cities in the country. Also, these teams are those with the highest average team value in the NBA. Therefore, we can name these groups as wealthiest teams.

The red group is the group with the teams that are less successful than the other teams. Between the 2013–2020 seasons, none of the teams played in the NBA finals. Moreover, none of the teams has championships except the Detroit Pistons and the Sacramento Kings. Therefore, this group can be named as teams with mediocre performance.

All teams in the gray group, except the Denver Nuggets, are on the east coast. This group includes teams with similar average team value and revenue. In addition, this group includes teams with a lower average attendence than other teams. So, this group can be named as less popular and teams with average team value. Finally, between the 2013–2020 seasons, all teams in the blue group, except the Dallas Mavericks and the Los Angeles Clippers, are the teams that have played the Conference final at least once. Furthermore, all teams in this group, played with an average attendence number of over 18,000. Thus, we can name this group as reliable teams.

**Fig 7:**
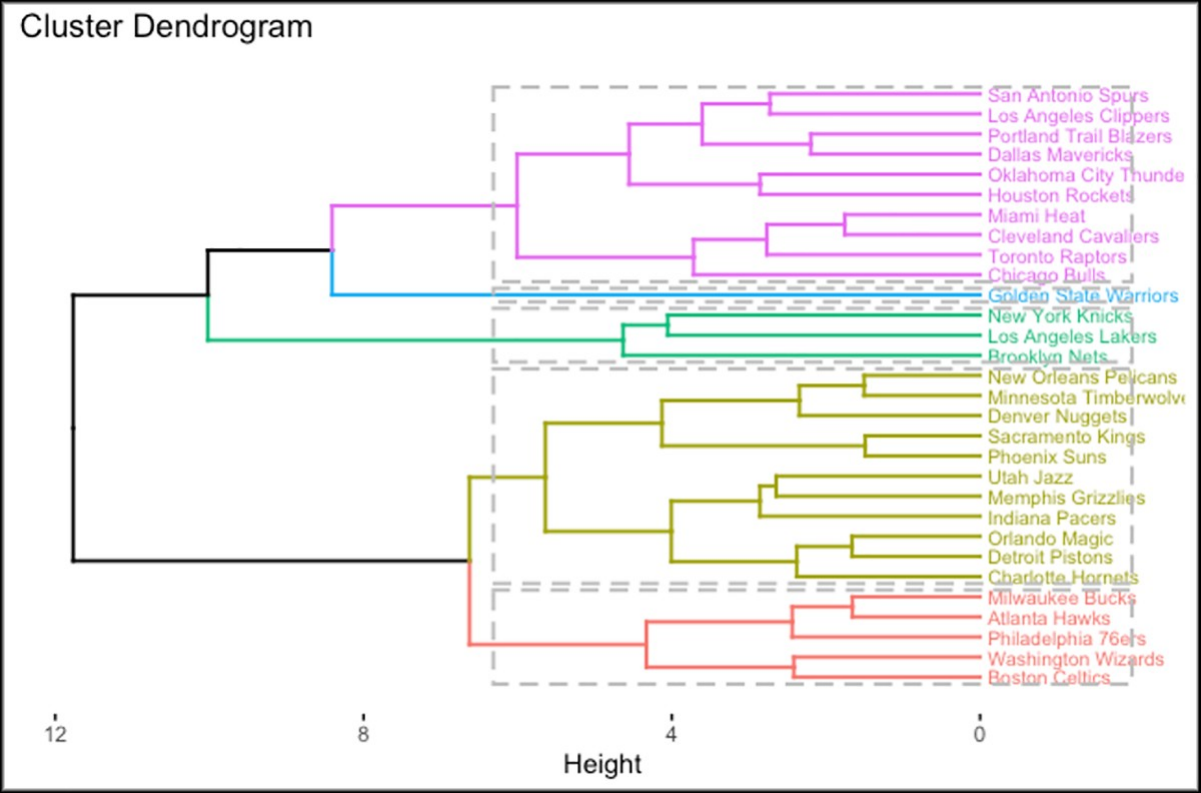
Plot of hierarchical clustering.

Alternatively, we can use other inspection method called Hierarchical clustering which does not require a pre-specified number of clusters. The algorithm of this approach considers all teams as single element clusters. Then, the most similar two clusters are combined into a new bigger cluster at each step. This process is repeated until all observations converge to one single cluster. Fig 7 illustrates the results of the Hierarchical Clustering.

Hierarchical clustering produced the similar results as K-means clustering. Generally, the teams are in the same groups as in the K-means clustering. Differently, the Chicago Bulls, Denver Nuggets and Indiana Pacers were included in different groups. This is because some teams are located on the boundaries of the groupings. Therefore, different clustering methods can assign some of the teams to other groups based on their location on the dendrogram.

### Linear models

Summary of the linear models are represented in Table 4 and seven different models; OLS, fixed effect model, linear fixed effect model, dummy variable fixed effect model, random effect model, linear random effect model, dummy variable random effect model, are included in the table. In Table 4, * denotes a statistically significant at significance level of 0.1, • represents a statistically significant at significance level of 0.05 and † shows a statistically significant at significance level of 0.01.

**Table 4:** Comparison of dynamic linear models.

Independent Variables	Dependent Variable: *Team Value*						
	OLS	FE	Linear FE	Dummy FE	RE	Linear RE	Dummy RE
Assist	6.878	3.806	0.676	2.815	4.289	2.32	4.289
Turnover	-26.9	-25.5	-2.88	-35.2	-9.81	9.85	-8.818
Points	0.869	3.348	12.17*	0.347	0.615	17.70†	0.615
Wining Percent	-2.85	-2.138	0.841	1.969	2.173	-0.006	2.173
Allstar	-52.441	-32.85	-14.381	-19.832	-38.3	-11.143	-38.322
Championship	32.056†	74.389	172.81†	46.269	31.44†	39.073†	31.440†
Population	0.00004†	0.001*	0.001•	0.001†			
GDP	0.007†	0.059†	0.042†	0.021*			
log(Population)					76.39•	134.973†	76.398•
log(GDP)					494•	566.011†	493.91•
Home Attendence	0.014	0.029	0.037*	0.062†	0.012	-0.010	-0.012
Revenue	8.372†	7.322†	4.408†	10.225†	8.90†	6.779†	8.902†
Coast	65.712				59.42		59.416
Season			119.512†				
Season 14				35.52			
Season 15				379.097†			
Season 16				308.34†			
Season 17				-148.886			
Season 18				-73.119			
Season 19				-104.272			
Season 20				116.805			
AIC	3406.45	3297.7	3268.8	3173.95	3360	3334.9	3360.01
Observations	239	239	239	239	239	239	239
R^2^	0.88	0.85	0.868	0.916	0.852	0.864	0.852
Adjusted R^2^	0.874	0.82	0.841	0.895	0.845	0.857	0.845

*: Statistically significant at significance level of 0.1.

•: Statistically significant at significance level of 0.05.

†: Statistically significant at significance level of 0.01.

The variables of the NBA data have characteristics that may vary from team to team. Therefore, OLS models cannot handle this issue. However, fixed effects models explore the relationship between dependent and independent variables within an entity and each entity has different characteristics that can affect the dependent variables. Fixed effects models help us to overcome this challenge. In contrast, random effects models assume the variation across and within entities are random and uncorrelated with the dependent or independent variables. Therefore, we assume fixed effects models estimate better results than other dynamic models for our NBA data. In Table 4, models are compared based on their AIC and R^2^ scores. The model which has the highest R^2^ and lowest AIC scores was selected as the winning model. It can be seen in Table 4 that dummy variable fixed effects model has the highest R^2^ and lowest AIC scores.

We also considered the models with lagged dependent variables in order to provide robust estimates of the effects of independent variables. Two different levels of lagged dependent variables specified in the model which accounts for auto-correlation in the error term. In Table 5, fixed effects models with one and two lags are compared with the winning model. Considering the lag of team value in the model helps us to explain the variation in the team value (for the certain year). Therefore, including the lags in the model yields more accurate parameter estimates. Based on the results in Table 4, the dummy fixed effects model with lag = 1 is selected as the final model. Including the lag value of dependent variable in the model overcomes the serial correlation issue. In the final model, five variables, i.e. Turnover, Championship, Population, GDP and Revenue are statistically significant.

**Table 5:** Comparison of fixed effects models.

Independent Variables	Dependent Variable		
	Dummy FE	Dummy FE (lag = 1)	Dummy FE (lag = 2)
Team Value(lag = 1)		0.662†	0.461†
Team Value(lag = 2)			-0.0052
Assist	2.815	11.331	9.013
Turnover	-35.171	-29.123•	-20.727•
Points	0.347	0.618	2.355
Wining Percent	1.969	1.402	1.161
Allstar	-19.832	14.561	16.349
Championship	46.269	52.964†	175.422†
Population	0.001†	0.0008•	0.001†
GDP	0.021*	0.01*	0.004
Home Attendence	0.062†	0.011	0.004
Revenue	10.225†	3.347†	4.081†
Season 14	35.520		
Season 15	379.097†		
Season 16	308.343†		
Season 17	-148.886		
Season 18	-73.119		
Season 19	-104.272		
Season 20	116.805		
AIC	3173.95	2029.35	2106.06
Observations	239	209	179
R^2^	0.916	0.967	0.961
Adjusted R^2^	0.895	0.957	0.947

*: Statistically significant at significance level of 0.1.

•: Statistically significant at significance level of 0.05.

†: Statistically significant at significance level of 0.01.

According to the final model, for team value at lag = 1, each one of Championship, Population, GDP, and Revenue has a significant positive effect on the team value while turnover has a negative effect. The coefficients of championship and revenue are highly significant on the team value. For instance, the team value increases by approximately $53 million for each championship. This result shows the important effect of the championship on team value. In addition, team value increases $3.347 million for a $1 million increases in the revenue. New York Knicks, Los Angeles Lakers, and Golden State Warriors have the highest average team values and those teams also have the highest revenues compared to other teams. Thus, our result supports the hypothesis that teams with the highest revenue and population tend to have the highest team value.

Turnover and population variables are statistically significant at the significance level of *α* = 0.05. Turnover has negative impact on the team value. Team value can be decreased by $52.9 million if the average turnover rises by one per game. For each additional 10,000 people in the population of the team city, the team value increases by $8 million. GDP is statistically significant at the significance level of *α* = 0.1. Each $100 increase in GDP rises the team value by $1 million. These results show that economic indices are more effective than performance variables in the estimation of team value. In other words, it is clear that a team value is highly affected by economic variables such as revenue and GDP. In the final model, the statistically significant variables on the team value at significance level of 0.1 found as: Turnover, Championship, Population GDP and Revenue. In addition, Championship and Revenue were also found statistically significant at significance level of 0.01 and Turnover was also found statistically significant at significance level of 0.05. Although it is found that the significant determinants of team value at the NBA team level are revenue, GDP, championship, and population, it is important to check the impact of the key player in the winning model. To do so, the predictor key player is added in the final model and compared with the winning model. The comparison of the final models is given in Table 6.

**Table 6:** Comparison of fixed effects models.

Independent Variables	Dependent Variable	
	Dummy FE (lag = 1)	Dummy FE (lag = 1)
Team Value(lag = 1)	0.662†	0.665†
Assist	11.331	11.352
Turnover	-29.123•	-28.251•
Points	0.618	1.514
Wining Percent	1.402	1.115
Allstar	14.561	10.648
Championship	52.964†	55.782†
Population	0.0008•	0.0004•
GDP	0.01*	0.01*
Home Attendence	0.011	0.006
Revenue	3.347†	3.225†
Key Player		4.953•
AIC	2029.35	1906.04
Observations	209	209
R^2^	0.967	0.974
Adjusted R^2^	0.957	0.962

*: Statistically significant at significance level of 0.1.

•: Statistically significant at significance level of 0.05.

†: Statistically significant at significance level of 0.01.

The team value is a very interesting metric to analyze because it encompasses almost all parts of a team. Although teams spent millions of dollars for a single key player, the key player may have a significant and positive impact on the team value. It can be seen from the Table 6 that key player is statistically significant on the team value. In the final model, we found that the significant determinants of team value at the NBA team level are revenue, GDP, championship, population, key player and turnover.

## Discussion

The main objective of this research is to examine the performance variables and economic indicators that significantly affect the team value. K-means clustering, hierarchical clustering, correlation, ordinary linear regression, fixed effect and random effect models were implemented to conduct the analyses. In correlation analysis, team value and revenue are found to be highly correlated with each other. In addition, there was a positive high correlation between GDP and team value. In contrast, home attendence and turnover have a negative relationship, high number of turnovers effect the number of home attendence in the teams. Unsurprisingly, winning percentage have a positive effect on the number of allstars selected from that team. Modeling has been done by taking these results into consideration in the further analysis.

For the cluster analysis, K-means and hierarchical clustering are applied to compare the teams in terms of overall impact. For clustering, 12 different variables were considered. Distance matrix of the variables were calculated to classify the teams into groups and to understand the characteristics of the overall impact. The optimal k was found to be 5 based on the gap statistic. Therefore, we divided the data into 5 clusters using K-means clustering and then performed the analysis with these results. Similarly, the hierarchical clustering divided the data into 5 clusters. The grouping of the teams was similar between the two approaches except for four teams; Chicago Bulls, Boston Celtics, Indiana Pacers and Denver Nuggets.

The dynamic regression models were considered in the modelling of the team values. Eight different models were compared based on AIC and *R*^2^ scores. The dummy variable fixed effects model was seen to be the optimal model in the first analysis. In all models, revenue was found to be the most significant variable on the team values. These finding are supported by other studies emphasizing the importance of revenue on the team value [3,4]. Furthermore, the three teams with the lowest average revenue in the NBA (2013–2020 seasons) are also the teams with the lowest team value.

Afterward, the models with lagged dependent variables are considered in order to provide robust estimates of the effects of independent variables and the final model was the dummy fixed effects model with lag = 1. According to the final model, each of Championship, Population, GDP, and Revenue has a significant positive effect on the team value while turnover has a negative effect. The coefficients of championship and revenue are highly significant on the team value. This means that the team which has the larger the number of championships, GDP, and revenue, tends to have the highest team values. This finding is consistent with similar studies [29,30].

Besides the economic variables affecting the team, there are also performance variables that have no significant effects on the team value. These include assists, points and winning percentages. Our findings, that relates the effect of the game performances on the team values, differs from the findings of [3]. The increase in the team value could be due to many indicators such as revenue, the number of championships, and so on. However, the impact of key players should be considered in the analysis. For example, the team value of the Cleveland Cavaliers increased by around 16% when the Lebron James joined the team first time. Similarly, the impact of his participation in Miami Heat helped increase the team value by about 10%. The team value of the Cleveland Cavaliers increased by over 70% after Lebron’s rejoined Cleveland Cavaliers in 2014. In addition, Lebron joined the Los Angeles Lakers in 2018 and the team value of the Los Angeles Lakers increased approximately by 34% in that season [21]. Similar scenarios were seen for the other key players. For instance, Kevin Durant has joined Golden State Warriors in 2016 and the team value of the GSW rose by around 27% in that year. It is not correct to say that the team value increased strictly only because of the key player. However, there is a strong correlation between the increase in the team value and key player. Our findings for the key player are consistent with other studies [1,21].

The other variables which may affect the team values, such as tv contracts of the teams, advertising agreements and player salaries, should be considered in the future studies. Although accessing such data is difficult and expensive, it would be worth the efforts to reach these data as these variables differ from team to team and may affect team values.

## Conclusion

The results of the cluster analysis help us to understand the similarities of the NBA teams based on the variables evaluated in this study. Thus, besides determining the parameters that affect the team value by using linear models, we aimed to investigate the NBA teams individually and examine their similarities with each other. Among all the teams, the results of the cluster analysis for Golden State Warriors (GSW) showed outstanding results and GSW distinguished from other teams. Furthermore, we were able to explain why some of the NBA teams with low performance statistics have the highest team value. In the yellow group of the cluster analysis, Brooklyn Nets, Chicago Bulls, Los Angeles Lakers and New York Knicks are the teams of the most three populous cities in America. Also, these teams are those with the highest average team value in the NBA. These results show that the NBA teams in the cities with a high population tend to have high team value. In the red group, there were teams with mediocre performance and none of the teams played in the NBA finals between the 2013 and 2020 seasons and only Sacramento Kings and Detroit Pistons have championships in the last 10 years. Therefore, the most of these teams have lower team values compared to yellow group. Examining the characteristics of NBA teams with cluster analysis using the selected variables helped us to understand the variables that affect team value in the mixed effect models.

In conclusion, this study clearly shows the importance of some economic variables and demographic indicators on team values. The most important variables on team values were revenue, key players, championship, and GDP. The level of the impact of those variables depends on the team. It is important to note that team value is not only influenced by the performance statistics. Other factors such as GDP and the population of the city should also be considered when invesigating team values in the analysis. It might be misleading to use only performance-based variables when analyzing the team values. Moreover, this study emphasizes the importance of analyzing the economic variables according to the team values. The results of the study could be valuable for owners and managers, but more research should be conducted addressing the impact of economic and demographic indicators on the team values. Our model could help managers and owners on their strategies to enhance team value and they can be prepared for different competitive scenarios.
